# Effect of retirement on cognitive function: the Whitehall II cohort study

**DOI:** 10.1007/s10654-017-0347-7

**Published:** 2017-12-26

**Authors:** Baowen Xue, Dorina Cadar, Maria Fleischmann, Stephen Stansfeld, Ewan Carr, Mika Kivimäki, Anne McMunn, Jenny Head

**Affiliations:** 1 Research Department of Epidemiology and Public Health, Institute of Epidemiology and Health Care, School of Life and Medical Sciences, University College London, London, UK; 2 Biostatistics and Health Informatics, Institute of Psychiatry, Psychology and Neuroscience, King’s College London, London, UK; 3 Department of Behavioural Science and Health, Institute of Epidemiology and Health Care, School of Life and Medical Sciences, University College London, London, UK; 4 Centre for Psychiatry, Wolfson Institute of Preventive Medicine, Barts and the London School of Medicine and Dentistry, Queen Mary University of London, London, UK

**Keywords:** Cognition, Retirement, Longitudinal study, Piecewise regression, Employment grade

## Abstract

According to the ‘use it or lose it’ hypothesis, a lack of mentally challenging activities might exacerbate the loss of cognitive function. On this basis, retirement has been suggested to increase the risk of cognitive decline, but evidence from studies with long follow-up is lacking. We tested this hypothesis in a cohort of 3433 civil servants who participated in the Whitehall II Study, including repeated measurements of cognitive functioning up to 14 years before and 14 years after retirement. Piecewise models, centred at the year of retirement, were used to compare trajectories of verbal memory, abstract reasoning, phonemic verbal fluency, and semantic verbal fluency before and after retirement. We found that all domains of cognition declined over time. Declines in verbal memory were 38% faster after retirement compared to before, after taking account of age-related decline. In analyses stratified by employment grade, higher employment grade was protective against verbal memory decline while people were still working, but this ‘protective effect’ was lost when individuals retired, resulting in a similar rate of decline post-retirement across employment grades. We did not find a significant impact of retirement on the other cognitive domains. In conclusion, these findings are consistent with the hypothesis that retirement accelerates the decline in verbal memory function. This study points to the benefits of cognitively stimulating activities associated with employment that could benefit older people’s memory.

**Electronic supplementary material:**

The online version of this article (10.1007/s10654-017-0347-7) contains supplementary material, which is available to authorized users.

## Introduction

Good cognitive functioning represents an essential element of healthy ageing and independent living [1]. There is some evidence that ageing affects cognitive functions that are primarily associated with executive processing and other functions of the frontal lobe [2, 3]. Thus, fluid abilities, such as memory, processing speed, and spatial ability tend to decline faster with age than crystallised functions, including vocabulary, information and comprehension [4–6]. However, the decline in these abilities is not necessarily homogenous across the population, as some people maintain cognitive vitality even into extreme old age [7–9]. On the one hand, there is evidence that the adult brain shows neuroplasticity and neurogenesis, representing the brain’s ability to generate new neurons and rewire itself [10–12]. On the other hand, accelerated deterioration or impairment in one or more cognitive functions beyond the ‘normal’ age-related decline could be predictive of the onset of dementia, a major cause of disability and dependency among older people worldwide [13, 14]. Therefore, it is important to identify and understand the predictors of interindividual differences in cognitive decline.

The theory of cognitive reserve proposes that some individuals have a larger cognitive reserve than others. It has been postulated that innate cognitive resources (such as childhood IQ), cognitive stimulation during brain maturation in childhood (such as education), and cognitively engaged lifestyle during adulthood (such as cognitively demanding occupation) can increase cognitive reserve, thus building up a buffer against cognitive decline in old age [15, 16]. The ‘use it or lose it’ hypothesis similarly suggests that a person can maintain cognitive function by engaging in cognitively demanding activities, whereas failing to keep mentally active will detrimentally affect cognitive function and could accelerate cognitive decline or even the onset of dementia [17]. Accordingly, retirement may be a potential trigger for cognitive decline, assuming that retirees leave paid work that is cognitively demanding. Many studies have supported this assumption showing that retirement is associated with lower cognitive functioning [18–23], and later retirement is associated with better cognition and lower risk of dementia [24–27], although some studies have found no association [28, 29] or even a positive effect [30] of retirement on levels of cognition.

When studying the effects of retirement on cognition, it is important to consider reverse causality. Declines in cognitive function may negatively affect the management of work tasks and thus could be a determinant of the decision to retire [31, 32]. For example, chronic diseases, such as stroke, might affect both cognitive function and retirement decisions [33, 34]. The vast majority of studies have compared retirees with working people to assess the potential effect of retirement on cognition, and it is possible that their results are biased due to a ‘healthy worker’ effect (i.e. people who remain in work are likely to be healthier than those who have stopped working). Some studies have relied on the use of instrumental variables, such as state pension age or early retirement windows, to eliminate bias due to unobserved heterogeneity and endogeneity [18–21, 28, 29]. The validity of the instrumental variables method relies on choosing an instrument that is not correlated with other factors that influence health and retirement. For example, in cross-national studies that have used state pension age as the instrument, it is possible that differences in state pension age may have been correlated with other national differences that affect health. A further limitation of these studies is their short follow-ups and use of only one measure of cognitive function. A few studies have examined change in cognitive function before and after retirement [22, 35], but most have relatively short follow-up period [36], and thus cannot estimate the long-term effects of retirement on cognitive function.

In this report from the Whitehall II cohort study, we compared trajectories of cognitive function before and after retirement within same persons up to 14 years before (mean 7.1 years) and 14 years after (mean 7.0 years) retirement, which included up to four repeated measures of cognitive function per person. This long period of follow-up helps reduce the possibility of reverse causation from health-related selection out of employment. To examine whether the ‘use it or lose it’ hypothesis applies to specific cognitive domains, we included several measures of verbal memory, abstract reasoning, phonemic verbal fluency, and semantic verbal fluency as cognitive outcomes. We further hypothesised that the influence of retirement on cognition may vary by socioeconomic status such that cognitive decline is more marked in individuals retiring from cognitively demanding higher employment grade jobs than in those who retire from less cognitively demanding low employment grade jobs [37]. Sex differences were also tested, because men and women may show advantages in different cognitive domains [38], and previous research has highlighted the gendered nature of employment trajectories and retirement [39], although men’s and women’s employment trajectories are becoming increasingly similar [40].

## Methods

### Study population and study design

This study used data from the Whitehall II prospective occupational cohort study. All civil servants aged 35–55 working in the London offices of 20 Whitehall departments in 1985–1988 were invited to participate. The response rate was 73% and a sample of 6895 men and 3413 women was recruited (phase 1). These civil servants were employed in a wide variety of roles from clerical grades, through to senior administrative grades, reflecting different employment grades and salaries. Follow-up surveys were conducted every 2–3 years. All participants provided written consent and the University College London ethics committee approved this study.

**Fig. 1:**
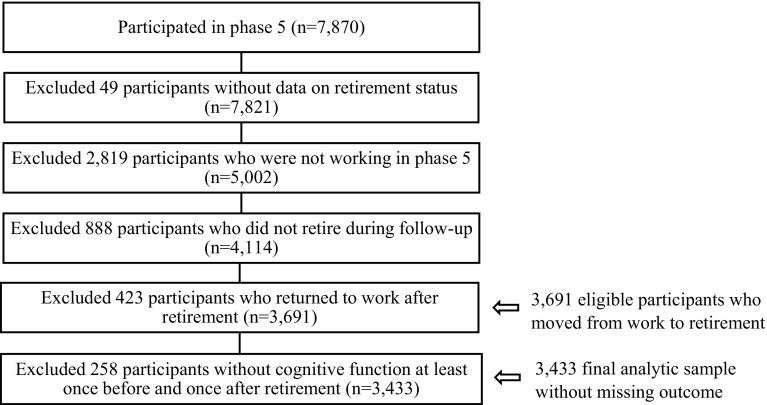
Flowchart of sample selection process

The data for the present analyses were drawn from phases 5 (1997–1999), 7 (2002–2004), 9 (2007–2009), and 11 (2012–2013) of the Whitehall II Study when cognitive tests were administered during the clinical examinations. Phase 3 (1991–1994) was not used because cognitive testing was introduced midway through phase 3 and consequently only half of respondents completed the cognitive test at that phase. For the current study, participants were eligible for inclusion if they had data on cognitive function at least once before and once after retirement. We excluded participants who were already not working at phase 5 and those who did not retire during follow-up or returned to work after retirement. There were 3691 eligible participants who moved from work to retirement, but 258 of these were excluded due to missing cognition outcome (i.e. without cognition measures at least once prior and at least once after retirement.) The final sample comprised 3433 participants (11,858 observations). The process of sample selection is shown in Fig. 1. Participants’ average age when taking the cognitive tests was 54.0 years (range 45–68) at phase 5, 59.5 years (range 51–74) at phase 7, 64.3 years (range 56–79) at phase 9, and 68.2 years (range 60–83) at phase 11.

### Measures

#### Cognitive function

The cognitive test battery, including verbal memory, abstract reasoning, phonemic verbal fluency, and semantic verbal fluency, was introduced to the Whitehall II cohort study in phase 5 and was repeated using the same tests at all subsequent assessments (phases 7, 9, and 11). The tests have good test–retest reliability (range 0.6–0.9), assessed in 556 participants who were invited back to the clinic within 3 months of having taken the test in phase 5 [41]. Verbal memory was assessed with a 20-word free recall test. Participants were presented with a list of 20 one- or two- syllable words at two-second intervals and then had 2 min to recall in writing as many words as possible (maximum possible score = 20) [42]. Abstract reasoning was assessed by the Alice Heim 4 Part 1 test (AH4). This test measures the ability to identify patterns and to infer principles and rules, which is composed of a series of 65 questions (32 verbal and 33 mathematical) of increasing difficulty (maximum possible score = 65). Participants had 10 min to complete this section [43]. Phonemic verbal fluency was assessed by asking participants to write as many words beginning with the letter ‘S’ as they could (maximum score = 35), and semantic verbal fluency was assessed by recalling as many animal names as possible (maximum score = 35). One minute was allowed for each verbal fluency test [44].

#### Retirement and year of retirement

Respondents’ employment status was measured by self-reports at each phase. Participants were considered to be in employment if they were still working in the civil service or were in paid employment elsewhere (full or part time). Participants were classified as retired if they moved from work to retirement directly or moved from work to unemployed/other, and then to retirement.

All respondents who retired from the civil service provided their exact year of exit from the civil service, but those who retired from employment outside the civil service were not asked the exact year of exit. For these 1632 individuals (46% of selected sample) whose exact exit year was unknown, we used the mid-point between the last phase still in paid work and the subsequent phase no longer working. We used the year of retirement as the centre point to calculate the cognitive trajectories before and after retirement.

#### Health-related retirement

At each phase, participants who were not working could indicate whether this was because of long-term sickness. Participants who retired from the civil service answered whether this was on health grounds. We considered participants who were ‘long-term sick’ or who indicated that the route of leaving the civil service was ‘retirement on health grounds’ as health-related retirement.

#### Covariates

We included retirement age as a covariate. Because all the analyses in this paper were centred at the year of retirement (see statistical method section), including retirement age as a covariate can effectively adjust for age effects. We adjusted for birth year to take account of the possibility of period effects. Gender and self-reported highest educational qualification were also included as covariates. Educational qualification was grouped into: O-level or lower (‘low’), A-level or equivalent (‘middle’), and degree level or higher (‘high’). To account for practice effects (i.e. gains in scores on cognitive tests when a person was retested on the same or similar instruments), we controlled for the number of cognitive tests a participant had completed in previous phases. Although cognitive test scores in phase 3 were not used in the analysis, the practice effect at this phase was counted.

Time-fixed covariates based on the last interview before retirement were employment grade, still working in the civil service, psychosocial job demands, job decision latitude, and spouse’s or partner’s employment status. Employment grade was measured, in order of increasing salary, as clerical/support (‘low’), professional/executive (‘middle’), or administrative (‘high’) [37]. For those who had left the civil service, the last employment grade before leaving was used. Job demands were measured by four items such as ‘Do you have to work very fast?’ Decision latitude was measured by nine items such as ‘Do you have a choice in deciding how to do your work?’ [45]. Respondents rated each question item whether it was ‘often’, ‘sometimes’, ‘seldom’ or ‘never/almost never’ the case. Each answer was scored from 0 to 3 and was added up so that a higher score reflected greater job demands or higher job decision latitude. Continuous scores were divided into tertiles [46]. Spouse’s employment status was measured by asking whether a spouse is currently doing any paid work. Those reporting not being married/cohabiting were coded as ‘no spouse’.

Time-varying covariates (phases 5, 7, 9, and 11) included smoking status, alcohol consumption, depressive symptoms, systolic blood pressure (SBP), diastolic blood pressure (DBP), body mass index (BMI), total blood cholesterol, coronary heart disease (CHD), stroke, all malignant cancers, and diabetes/intermediate hyperglycaemia. By treating these variables as time-varying, we account for reported changes in health conditions and health behaviours over time. Smoking status (current, never, ex-smoker) and alcohol consumption in the past week (0, 1–10, more than 10 units) were based on self-reports. Symptoms of depression were measured by the depression subscale of the General Health Questionnaire (GHQ), and cut-off points of four out of 12 were used to identify depression cases [47]. Blood pressure (mm Hg), BMI (kg/m^2^), and total blood cholesterol (mmol/l) were objectively measured during the clinical examinations and were included as continuous covariates in the model. CHD (yes/no) includes diagnosed non-fatal myocardial infarction (MI) and ‘definite’ angina. Non-fatal MI was defined following MONICA criteria [48] based on study electrocardiograms, hospital acute ECGs, and cardiac enzymes and validated using discharge diagnoses from National Health Service (NHS) Hospital Episode Statistics (HES) data or General Practitioner (GP) confirmation up to the end of phase 11. Self-reports of non-fatal MI were not used [49]. ‘Definite’ angina included self-reported cases of angina only if they were subsequently validated by these other sources. Self-reported stroke events (yes/no) were collected throughout follow-up, and were validated by HES data linkage, GP’s confirmation, or retrieval of hospital medical records up to phase 9 [49, 50]. Cancer incidence data (yes/no) for the period 1971–2015 were obtained from the NHS Central Register for nearly all participants. Diabetes/intermediate hyperglycaemia (yes/no) was defined by the WHO criteria of oral glucose tolerance test and by a self-reported diagnosis of diabetes [51].

### Statistical methods

To test a change of the response function (Y) of a varying independent variable (X), we used piecewise linear regression with two segments separated by a ‘knot’ [52, 53]. We used year of retirement as the knot (i.e. year 0), and thus, generated two independent variables reflecting ‘years before retirement’ (− 14 to − 1) and ‘years after retirement’ (1–14). Retired less than a year was counted as 1 year. Linear mixed models were fitted for each cognition outcome, in turn, and these two variables were entered into the model. The coefficients for the variable ‘years before retirement’ (i.e. slope before) represented the average change in cognition per year before retirement. Coefficients for the variable ‘years after retirement’ (i.e. slope after) represented the average change in cognition for each additional year after retirement. If retirement did not affect cognition, we would expect the trajectories of cognitive function to be similar before and after retirement. Therefore, to test whether retirement influenced cognitive decline, independent of age-related change, we examined differences in the slope for cognition before and after retirement. The ‘slope change’ was defined as the ‘slope after retirement’ minus the ‘slope before retirement’ (this was also expressed as percentage change, calculated as ‘slope change’ divided by slope before retirement multiplied by 100). All analyses were carried out in Stata 14. We also examined whether a nonlinear piecewise model was better than the linear model by adding quadratic terms of ‘years before retirement’ and ‘years after retirement’ into each model. To take account of the clustering of the data, mixed models with repeated measures and individuals as the two random-effects levels were conducted. The models allowed for both random intercepts (for each individual) and random coefficients (for the terms ‘years before’ and ‘years after’ retirement).

To assess whether the effect of retirement differed by cognition domains, we conducted a test of heterogeneity on the effect of retirement using multivariate multilevel models with all cognition outcomes included in one model.

To visualise the results from these regressions, we show predicted trajectories of each cognitive function outcome, both before and after retirement. These predicted trajectories from adjusted models were calculated at the sample mean of each covariate. In addition to piecewise linear trajectories (where ‘years before retirement’ and ‘years after retirement’ were treated as continuous), predicted adjusted means at each time point (where ‘years before retirement’ and ‘years after retirement’ were treated as categorical) are shown as dots in the figures.

We tested for potential moderators, including employment grade (based on last response before retirement) and sex in the association between retirement and cognition outcomes, by adding interaction terms (‘years before retirement × employment grade’ and ‘years after retirement × employment grade’; ‘years before retirement × sex’ and ‘years after retirement × sex’) in the model for each cognition outcome.

### Missing data

For time-fixed covariates (employment grade, still working in the civil service, job demands, job decision latitude, and partner’s employment status), missing data in the last interview before retirement was first replaced by prior responses. The remaining missing data of time-fixed covariates and missing data of other covariates for the eligible participants were imputed in Stata, using multivariate imputation by chained equations, and 30 datasets were imputed. We included all variables from the analyses (i.e. independent variables, outcome variables, covariates, and moderators) in the imputation model. After running the imputation, we deleted imputed outcome values in the regression. Percentage of missing data was shown in Table 1.

**Table 1:** Descriptive statistics for the study sample (n = 3433)^a^

	Observed N (% missing)	Observed %	Imputed %	Observed mean (SD)	Imputed mean (SD)
Retirement age	3433 (0%)			61.2 (4.6)	61.2 (4.6)
Verbal memory	3433 (0%)			6.9 (2.3)	6.9 (2.3)
Abstract reasoning	3433 (0%)			45.4 (11.0)	45.4 (11.0)
Phonemic verbal fluency	3433 (0%)			16.0 (3.9)	16.0 (3.9)
Semantic verbal fluency	3433 (0%)			16.3 (4.1)	16.3 (4.1)
Birth year	3433 (0%)			1943.7 (5.0)	1943.7 (5.0)
Gender	3433 (0%)				
Men		72.2	72.2		
Highest education qualification	3323 (3.2%)				
O level or lower		41.7	42.0		
A level or equivalent		24.8	24.7		
Degree level or higher		33.5	33.3		
Employment grade	3433 (0%)				
Clerical/support (lowest)		12.1	12.1		
Professional/executive		42.2	42.2		
Administrative (highest)		45.7	45.7		
Still in civil service	3400 (1.0%)	62.0	62.1		
Job demand	3433 (0%)				
Low		24.4	24.4		
Middle		46.2	46.2		
High		29.4	29.4		
Job decision latitude	3433 (0%)				
Low		24.8	24.8		
Middle		31.7	31.7		
High		43.5	43.5		
Spouse’s employment status	3341(2.7%)				
Working spouse		50.3	50.1		
Non-working spouse		28.3	28.3		
No spouse		21.4	21.6		
Smoking status	3312 (3.5%)				
Never-smoker		49.0	48.9		
Ex-smoker		41.6	41.8		
Current smoker		9.4	9.3		
Alcohol consumption	3293 (4.1%)				
None last week		15.0	15.1		
≤ 10 units last week		42.1	42.0		
> 10 units last week		42.9	42.9		
GHQ depression (≥ 4)	3300 (3.9%)				
Yes		11.4	11.5		
BMI^b^	3068 (10.6%)			26.6 (4.2)	26.7 (4.2)
Normal (< 25 kg/m^2^)		37.5	37.2		
Overweight (25–29.9 kg/m^2^)		44.4	44.3		
Obese (≥ 30 kg/m^2^)		18.1	18.5		
Blood pressure^b^	3232 (5.9%)			124.6 (16.0)^c^	124.7 (16.0)^c^
High (≥ 140/90 mmHg)		18.7	18.9	75.0 (10.7)^d^	75.1 (10.7)^d^
Blood cholesterol^b^	3200 (6.8%)			5.7 (1.0)	5.7 (1.0)
High (≥ 5 mmol/l)		77.0	77.1		
CHD	3433 (0%)	7.5	7.5		
Stroke	3433 (0%)	0.3	0.3		
Cancer	3400 (0.1%)	3.5	3.5		
Diabetes	3433 (0%)	21.6	21.6		

^a^Information for the cognitive outcomes and time-varying covariates (smoking status, alcohol consumption, GHQ depression, BMI, blood pressure, blood cholesterol, CHD, stroke, cancer, diabetes) were based on the last interview before retirement

^b^BMI, blood pressure, and blood cholesterol were used as continuous variables in regression models

^c^Mean (SD) for systolic blood pressure

^d^Mean (SD) for diastolic blood pressure

### Sensitivity analyses

We conducted three sensitivity analyses to assess the reliability of our results and conclusions. Sensitivity analysis 1 aimed to assess potential bias to the results due to reverse causality. For this analysis, we excluded 500 participants from the analytic sample who retired due to health reasons or had a GHQ depression value of 4 or higher at the last interview before retirement. In addition, 172 participants for whom cognition was measured only twice (once before and once after retirement) were also excluded. Participants who moved from work to retirement via ‘unemployed/other’ (n = 278) were excluded from this sensitivity analysis, since they are likely to have higher levels of stress which may influence cognitive function. Some participants fulfilled several of the exclusion criteria, thus a total of 911 participants were excluded in this sensitivity analysis.

We compared the characteristics of ‘eligible participants but with missing cognition data (n = 258)’ and ‘the analytic sample (n = 3433)’, and found that they had several different demographic characteristics (online resources Table 1S). Therefore, it is possible that our analytic sample had different cognitive function compared to participants with missing cognitive data. Sensitivity analysis 2 aimed to assess the impact of missing cognitive data on results. This sensitivity analysis included these 258 participants and multiply imputed their missing cognitive measures.

Sensitivity analysis 3 assessed whether our results could be influenced by physical activity level, although one recently published Whitehall II study found no association between physical activity and cognitive decline [54]. We used the total physical activity level (< 8, 8–12, ≥ 12 h/week) at the last interview before retirement.

## Results

Table 1 shows that the analytical sample includes 3433 participants of whom 72% were men. Their average retirement age was 61.2 years (SD = 4.6), which is slightly higher than the civil service occupational pension age (60 years). Descriptive information for the cognitive outcomes and time-varying covariates refers to the last interview before retirement. Most respondents’ (66.5%) highest educational qualification was lower than degree level. Most respondents were employed in the highest (‘administrative’, 45.7%) or second highest (‘professional/executive’, 42.2%) employment grade, and 62% of respondents were still working in the civil service (rather than working outside). 50.3% had a working spouse while 21.4% did not have a spouse. Nearly half of the sample were never-smokers, and 15% did not consume any alcohol in the past week. Eleven percent of respondents had a raised GHQ depression score. Approximately, one in five had hypertension, 77% had elevated levels of blood total cholesterol, and nearly three out of five were overweight (44.4%) or obese (18.1%). Imputed data showed very similar percentages and means as observed data.

Table 2 shows the fully adjusted models on retirement and cognitive function from piecewise linear regressions. The negative pre-retirement slope for verbal memory (− 0.103; 95% CI − 0.122, − 0.085) suggests that verbal memory was inversely associated with ‘years before retirement’, or in other words, verbal memory score declined by 0.103 every year before retirement. After retirement, verbal memory scores declined by 0.143 every year (95% CI − 0.162, − 0.124). The slope change (the difference between after vs. before retirement cognition slopes) was − 0.039 (95% CI − 0.058, − 0.021; *p* < 0.001), indicating that verbal memory test scores declined faster after retirement compared to before retirement. The % change was 38% [calculated as: $\left( { - \,0.039 \div - \,0.103} \right) \times 100\%$], suggesting that retirement was associated with, on average, 38% faster decline in verbal memory, independent of age-related decline. The results for other cognition domains (abstract reasoning, phonemic and semantic verbal fluency) showed that while there was age-related decline in cognitive function both before and after retirement, the differences in the slope of decline were not statistically significant at conventional levels (*p* = 0.180 for abstract reasoning; *p* = 0.867 for phonemic verbal fluency; *p* = 0.774 for semantic verbal fluency). Adding quadratic terms in the piecewise regressions did not improve model fit (Wald test *p* > 0.1, results are not shown). In the multivariate multilevel analysis including all cognitive domains, the test of heterogeneity confirmed that the effect of retirement on verbal memory was more pronounced compared to other domains of cognition (verbal memory vs. abstract reasoning *p* = 0.001; verbal memory vs. phonemic verbal fluency *p* = 0.028; verbal memory vs. semantic verbal fluency *p* = 0.070).

**Table 2:** Changes of cognition trajectories before and after retirement (n = 3433)^a^

	Before retirement			After retirement			Change^b^			
	Slope (change per year)	95% CI	*p*	Slope (change per year)	95% CI	*p*	Slope (change per year)	95% CI	*p*	% change (%)^c^
Verbal memory	− 0.103	− 0.122, − 0.085	< 0.001	− 0.143	− 0.162, − 0.124	< 0.001	− 0.039	− 0.058, − 0.021	< 0.001	38.0
Abstract reasoning	− 0.579	− 0.643, − 0.513	< 0.001	− 0.547	− 0.616, − 0.477	< 0.001	0.032	− 0.015, 0.078	0.180	5.5
Phonemic verbal fluency	− 0.219	− 0.250, − 0.189	< 0.001	− 0.217	− 0.249, − 0.184	< 0.001	0.002	− 0.025, 0.029	0.867	0.9
Semantic verbal fluency	− 0.168	− 0.196, − 0.139	< 0.001	− 0.164	− 0.194, − 0.134	< 0.001	0.004	− 0.022, 0.029	0.774	2.3

^a^Adjusted for retirement age, birth cohort, highest educational qualification, gender, practice effects, spouse employment status, employment grades, still working in the civil service, job demands, job decision latitude, smoking status, alcohol consumption, depressive symptoms, systolic blood pressure, diastolic blood pressure, body mass index, total blood cholesterol, coronary heart disease, stroke, malignant cancers, and diabetes/intermediate hyperglycaemia

^b^Calculated as ‘slope after retirement’ minus ‘slope before retirement’

^c^Calculated as ‘slope change’ divided by ‘slope before retirement’ and multiplied by 100%

**Fig. 2:**
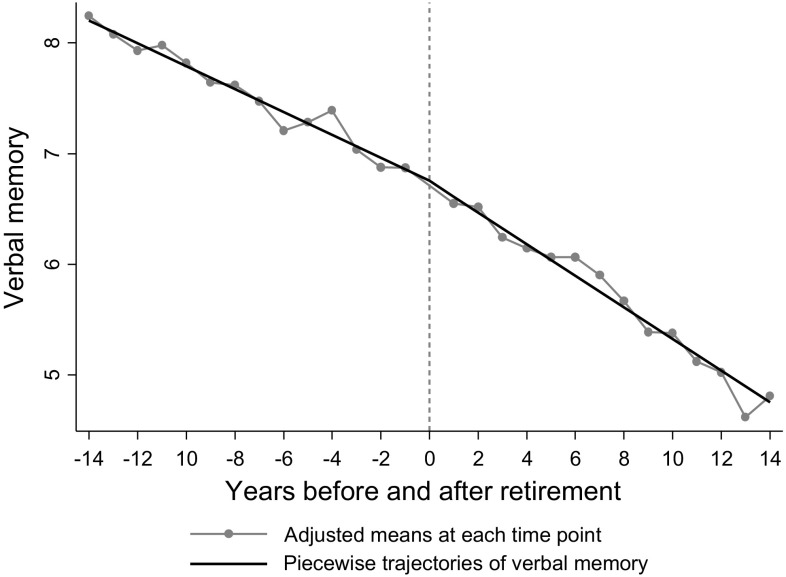
Trajectories of verbal memory before and after retirement by averaging covariates

**Fig. 3:**
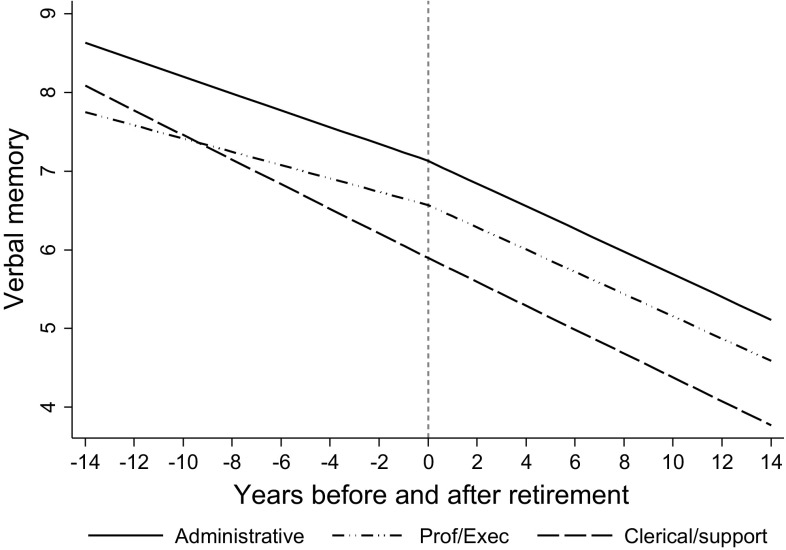
Trajectories of verbal memory by employment grade

To visualise the results of verbal memory, trajectories of adjusted means, both before and after retirement, are shown in Fig. 2. As explained above, the decline in verbal memory accelerated after, compared to before, retirement. The trajectories for the other three domains are shown in the online resources (Figs. 1S to 3S), because there was no significant difference in the decline before and after retirement.

We then examined whether the association between retirement and cognitive function varied by employment grade. Including terms for the interaction between employment grade and slopes for verbal memory revealed that the interaction was borderline significant (Wald test *p* = 0.062, results are not shown). For better interpretation, we stratified the models for verbal memory by employment grade. Stratified results in Table 3 show that pre-retirement slopes were less negative among those with higher employment grades (− 0.084 for professional/executive, − 0.107 for administrative) compared to those in the clerical/support grade (− 0.156), suggesting that higher employment grade may be protective against verbal memory decline while people were still working. This ‘protective effect’ disappeared when individuals retired, as people had similar slopes of verbal memory after retirement, which was − 0.152 for clerical/support grade, − 0.142 for professional/executive grade, and − 0.144 for administrative grade. Those retiring from professional/executive (slope change = − 0.057; 95% CI − 0.086, − 0.029; % change = 67.9%) and administrative grades (slope change = − 0.037; 95% CI − 0.063, − 0.011; % change = 34.6%) experienced significant changes in their slopes of verbal memory, but those retired from the clerical/support grade did not. Figure 3 plots trajectories of verbal memory by employment grade. This highlights that people in higher grades start out with better verbal memory (i.e. higher intercept) and a slower rate of decline (i.e. less negative slope) while in work. After retirement, rates of decline were similar across employment grades, although verbal memory level remains higher among participants from higher employment grades. The interaction between sex and slopes for verbal memory was not statistically significant (Wald test *p* = 0.31), thus models were not stratified by sex.

**Table 3:** Stratified results for verbal memory by employment grade (n = 3433)^a^

	Before retirement			After retirement			Change^b^			
	Slope (change per year)	95% CI	*p*	Slope (change per year)	95% CI	*p*	Slope (change per year)	95% CI	*p*	% change (%)^c^
Clerical/support(low)	− 0.156	− 0.217, − 0.096	< 0.001	− 0.152	− 0.210, − 0.094	< 0.001	0.005	− 0.064, 0.073	0.893	3.2
Prof/exec(middle)	− 0.084	− 0.112, − 0.057	< 0.001	− 0.142	− 0.171, − 0.112	< 0.001	− 0.057	− 0.086, − 0.029	< 0.001	67.9
Administrative(high)	− 0.107	− 0.134, − 0.080	< 0.001	− 0.144	− 0.173, − 0.116	< 0.001	− 0.037	− 0.063, − 0.011	0.005	34.6

^a^Adjusted for retirement age, birth cohort, highest educational qualification, gender, practice effects, spouse employment status, still working in the civil service, job demands, job decision latitude, smoking status, alcohol consumption, depressive symptoms, systolic blood pressure, diastolic blood pressure, body mass index, total blood cholesterol, coronary heart disease, stroke, malignant cancers, and diabetes/intermediate hyperglycaemia

^b^Calculated as ‘slope after retirement’ minus ‘slope before retirement’

^c^Calculated as ‘slope change’ divided by ‘slope before retirement’ and multiplied by 100%

Three sensitivity analyses did not change the associations between retirement and cognitive outcomes. Results are shown in online resources Tables 2S to 4S.

## Discussion

In this longitudinal study of 3433 individuals, we compared cognitive decline before and after retirement and examined whether trajectories varied depending on employment grade. We found that declines in verbal memory were faster during the 14 years after than during the 14 years before retirement. In the stratified analysis, we found that higher employment grade may be protective against verbal memory decline while people were still working, but this ‘protective effect’ was lost when individuals retired. The other domains of cognitive function, including abstract reasoning, phonemic verbal fluency, and semantic verbal fluency, were not affected by retirement, but declined steadily with age.

Our finding showing an adverse effect of retirement on verbal memory is consistent with most previous studies which used instrumental variables [18–21] and also those which have applied other statistical methods. For instance, Wickrama and O’Neal [35] used growth curve analyses in the USA Health and Retirement Study (HRS), and found that individuals who retired between 1998 and 2002 had a faster memory decline between 2002 and 2006 compared to those who were working at both instances. In another HRS investigation, Clouston and Denier [22] showed similar findings for retirement and episodic memory (comprised of both verbal learning and verbal memory) by using longitudinal regression discontinuity methods to analyse trajectories between 1998 and 2012. A cross-national study by Adam et al. [55] used the stochastic frontier approach to estimate the episodic memory that individuals would reach if they were fully efficient for a given level of resources. They found an adverse effect of retirement on episodic memory and highlighted the positive impact non-professional activities at retirement and increased social contacts could have for episodic memory.

Our finding on the retirement-associated decline in verbal memory supports the ‘use it or lose it’ hypothesis suggesting that failing to keep mentally active may accelerate the rate of cognitive decline in post-retirement periods [17]. On a similar note, our findings are also consistent with the theory of ‘mental retirement’ proposed by Rohwedder and Willis [21], suggesting that the work environment could be more cognitively stimulating than the leisure environment as a retiree. Besides the direct effects of an absence of cognitive activities related to work, retirement may also affect cognitive function indirectly via loss of work-related forms of self-organisation, communications and collaborations [56], which are important factors potentially contributing to the maintenance of cognitive reserve [55, 57]. For example, social networks could be more extensive during employment, and accordingly, Börsch-Supan and Schuth [58] estimated that at least one-third of the decline in cognition after retirement could be attributed to a reduction in the size and composition of social networks.

Our results showing a significant effect of retirement for verbal memory but not for other cognitive domains suggest that retirement may affect some cognitive domains more than others. Age-related neuronal modifications that are at the root of Alzheimer’s disease have been observed to have heterogeneous effects on cognitive functioning. For example, episodic memory deficits are largely considered as a hallmark symptom of Alzheimer’s disease [59], but this is less the case for other domains of cognition. It may also be that verbal memory is a more sensitive indicator of cognitive decline than the other indicators. Few studies have assessed the effect of retirement on different domains of cognition. Using the USA Wisconsin Longitudinal Study, Denier et al. [60] found that those who had retired voluntarily or for family reasons had improved reasoning abilities, which is contrary to our findings. Denier et al. used the similarities construct of the Weschler Adult Intelligence Scale to measure reasoning abilities. Respondents were asked to relate two words; for instance, ‘How are an apple and orange alike?’ to which they should respond that both are fruits. In contrast, the AH4 questionnaire used in our study consists of both verbal and mathematical questions. Inconsistent results may originate from different assessments of cognitive functioning across studies. One SHARE study tested only memory and numeracy and found that both domains were negatively affected by retirement [58]. Also using SHARE data, Mazzonnaa and Peracchi found that retirement was negatively associated with verbal memory, orientation, and numeracy for both men and women. Retirement was not associated with verbal fluency, with the exception that retired women without a high-school degree showed a faster decline in verbal fluency [20]. Our Whitehall II study did not measure orientation or numeracy, but our results of the negative effect on memory and no influence on verbal fluency are generally consistent with these two SHARE studies. Roberts et al. [36] previously used Whitehall II to show that mean cognitive test scores increased between two assessments over 5 years, and discussed that this is possibly due to practice effects. They found that those retired increased less than those still working. Their findings could not be confirmed by our study using longer follow-up of Whitehall II, where we have taken account of practice effects by adjusting for the number of cognitive tests a participant has completed in previous phases.

Stratified analyses showed that higher employment grade may be protective against cognitive decline while people were still working, but this ‘protective effect’ went away when individuals retired. According to the cognitive reserve hypothesis, engagement in mentally challenging activities can yield additional neuronal resources that may prevent cognitive decline [57]. Higher grade jobs have higher levels of skill discretion implying more opportunities for the use of skills and variety of work [37], which suggests higher levels of mental processing than clerical/support jobs. Thus, our observation that employees in higher grades had slower decline during employment is plausible. This protection of higher grades no longer exists after retirement. As expected by the ‘use it or lose it’ hypothesis, the decline in verbal memory was similar in all participants irrespective of their pre-retirement employment grade.

Our findings on employment grade are in agreement with the observations from SHARE, showing that the average effect of retirement on cognition was negative, and the negative effect of retirement disappeared when the sample was restricted to people who worked in more physically demanding occupations [61]. Our stratified results are also in line with Finkel et al.’s work, which found that retirement from more complex jobs was related to a faster rate of cognitive decline in the longitudinal Swedish Adoption/Twin Study of Aging [62]. However, Fisher et al. [30] found that participants from occupations characterised by higher levels of mental demands showed slightly higher cognitive performance and less steep decline both before and after retirement, compared with individuals who were engaged in fewer mental demands. It is likely that employment grades may not only represent job mental demands but also serve as an indicator of broader working environments as well as post-retirement financial resources and social support. People in higher employment grades may have a stronger attachment to their work role, and thus retirement may be more detrimental to them because of this role loss. Future studies might investigate different preretirement occupational characteristics in order to understand the nature and mechanisms underlying the cognitive effects of retirement.

Our findings on employment grade should be interpreted cautiously because only 12.1% of our analytic sample was retired from clerical/support grade, and the interaction by employment grade was only marginally significant. Therefore, we cannot rule out the possibility that the different results found for clerical/support grade were due to selection bias. It is also worth pointing out that, even though individuals in higher grades had a faster rate of cognitive decline after retirement compared to before retirement, they still had higher average levels of cognition than people in the lowest grade, both before and after retirement. This suggests that although retirement seems to be more detrimental for those in higher grades, people in the lowest grade remain at greatest risk of developing cognition problems.

We found no significant sex differences in terms of the effect of retirement on verbal memory. However, less than 30% of our sample are women, with even fewer women in the higher grades, so it is possible that the study lacked the power to detect potential sex differences.

### Strengths and limitations

The main strength of our study is the assessment of multiple cognitive domains and a long observation period both before and after retirement. To our knowledge, this is the first study on cognition with such extended periods of pre- and post-retirement measures.

It is also a strength that we could examine the rate of change in cognition before and after retirement rather than comparing levels of cognition. Cognitive decline could lead to retirement but the analysis reduced this problem by comparing slopes from long follow-up before and after for the same group of individuals. This way, our method could lower the risk of reverse causality. The consistent findings in the sensitivity analysis reassured us of our results. The use of a multilevel framework in this study was able to account for clustering of observations within participants. Missing values in the covariates were multiply imputed using chained equations, allowing us to use information from all cases. Predicted piecewise cognition trajectories showed the trajectories before and after retirement.

There are also several limitations to this study. First, a great challenge with this type of research is the possibility of reverse causality. Although this piecewise study has several strengths in attempting to reduce the influence of reverse causality, the possibility of reverse causality is still not fully eliminated. Loss-to-follow-up bias is also possible because we only included retired participants with repeated observations both before and after retirement. Some participants dropped out of the study earlier than others, and thus had fewer repeated measures of cognition, which may lead to nonignorable missingness. Also, the level of cognitive function was measured at every other wave rather than at each wave, which increased the likelihood of loss-to-follow-up for older participants. We adjusted for health conditions that are related to cognitive decline, but confounding from unknown characteristics is still possible. Random intercepts and random coefficients accounted for individual trajectories of cognitive function before and after retirement and captured such unobserved variability to some extent. However, even though the proportion of participants who died or were diagnosed with dementia is very small in this cohort, results may be biased by accelerating declines of cognition occurring before dementia [63] or prior to death [64, 65]. Our study focuses on the average slope change of cognitive function as a response to retirement. We did not examine whether post-retirement activities, such as voluntary work, social activities, and physical activities may modify the risk of cognitive decline. Further research may focus on factors explaining heterogeneity in declines in cognitive functioning after retirement.

The Whitehall II Study uses a sample of civil servants in the UK. Compared to the general population, their type of work may be more mentally challenging. For example, verbal memory may be especially important for their paperwork. Thus, their cognition trajectories cannot be regarded as being representative of the general population, although the sample covers the entire range of occupations from administrative to clerical/support, ensuring some level of variability. Moreover, the sample size at the two ends of the analytic period is smaller than those at other time points, leading to larger confidence intervals at the two ends. However, the estimated coefficients of our piecewise model are based on the overall linear trend of cognition, and showed a good fit between the estimated line and observed values at each time point.

## Conclusion

In support of the ‘use it or lose it’ hypothesis, we found that retirement is associated with faster declines in verbal memory function over time, but has little impact on other domains of cognitive functions, such as abstract reasoning and verbal fluency. The smaller cognitive decline before retirement in employees from high employment grade jobs points to the potential benefits of cognitively stimulating activities associated with employment that could benefit older people’s memory.

## Electronic supplementary material

Below is the link to the electronic supplementary material.

Supplementary material 1 (DOCX 38 kb)
