# Supplementary material for: Effect of retirement on cognitive function: the Whitehall II cohort study
Source: Eur J Epidemiol. 2017 Dec 26;33(10):989–1001. doi: 10.1007/s10654-017-0347-7 (PMC6153553; doi:10.1007/s10654-017-0347-7)
Supplement: Supplementary file 1 — Supplementary material 1 (DOCX 38 kb) [file 10654_2017_347_MOESM1_ESM.docx]

**Figure 1S. Trajectories of abstract reasoning**

**Figure 2S. Trajectories of phonemic verbal fluency**

**Figure 3S. Trajectories of semantic verbal fluency**

**Table 1S. Comparing characteristics between excluded eligible participants due to missing cognition and the final analytic sample.**

|  | **Excluded eligible participants due to missing cognition ^a^** **(n=258)** | **Final analytic sample (n=3433)** | ***p* for Chi^2^** |
| --- | --- | --- | --- |
| Retirement age | 60.6 | 61.2 | 0.060 |
| Birth year | 1943.0 | 1943.7 | 0.030 |
| Gender |  |  |  |
| Men | 63.2 | 72.2 | 0.002 |
| Highest education qualification |  |  |  |
| O level or lower | 44.9 | 41.7 | 0.645 |
| A level or equivalent | 23.1 | 24.8 |  |
| Degree level or higher | 32.0 | 33.5 |  |
| Employment grade |  |  |  |
| Clerical/support (lowest) | 29.8 | 12.1 | <0.001 |
| Professional/executive | 33.3 | 42.2 |  |
| Administrative (highest) | 36.8 | 45.7 |  |
| Still in civil service | 60.8 | 62.0 | 0.721 |
| Job demand |  |  |  |
| Low | 29.3 | 24.4 | 0.200 |
| Middle | 42.2 | 46.2 |  |
| High | 28.5 | 29.4 |  |
| Job decision latitude |  |  |  |
| Low | 35.3 | 24.8 | 0.001 |
| Middle | 26.0 | 31.7 |  |
| High | 38.8 | 43.5 |  |
| Spouse’s employment status |  |  |  |
| Working spouse | 48.8 | 50.3 | 0.919 |
| Non-working spouse | 29.1 | 28.3 |  |
| No spouse | 22.2 | 21.4 |  |
| Smoking status |  |  |  |
| Never-smoker | 39.1 | 49.0 | <0.001 |
| Ex-smoker | 41.7 | 41.6 |  |
| Current smoker | 19.1 | 9.4 |  |
| Alcohol consumption |  |  |  |
| None last week | 23.5 | 15.0 | 0.050 |
| ≤10 units last week | 36.5 | 42.1 |  |
| >10 units last week | 40.0 | 42.9 |  |
| GHQ depression (≥4) |  |  |  |
| Yes | 11.6 | 11.4 | 0.828 |
| BMI ^b^ |  |  |  |
| Normal (<25 kg/m^2^) | 39.7 | 37.5 | 0.884 |
| Overweight (25-29.9 kg/m^2^) | 43.8 | 44.4 |  |
| Obese (≥30 kg/m^2^) | 16.4 | 18.1 |  |
| Blood pressure ^b^ |  |  |  |
| High (≥140/90mmHg) | 24.7 | 18.7 | 0.133 |
| Blood cholesterol ^b^ |  |  |  |
| High (≥ 5mmol/l) | 81.6 | 77.0 | 0.158 |
| CHD | 7.3 | 7.5 | 0.849 |
| Stroke | 0.4 | 0.3 | 0.709 |
| Cancer | 4.7 | 3.5 | 0.622 |
| Diabetes | 14.1 | 21.6 | 0.004 |

^a^ Eligible participants but without cognition measures at least once before and once after retirement**.**

**Table 2S.** **Changes of cognition trajectories before and after retirement from sensitivity analysis 1 (n=2,522) ^a^**

|  | **Before retirement** | | |  | **After retirement** | | |  | **Change ^b^** | | | |
| --- | --- | --- | --- | --- | --- | --- | --- | --- | --- | --- | --- | --- |
|  | **Slope**  **(change per year)** | **95%CI** | ***p*** |  | **Slope**  **(change per year)** | **95%CI** | ***p*** |  | **Slope**  **(change per year)** | **95%CI** | ***p*** | **%**  **change ^c^** |
| Verbal memory | -0.099 | -0.121, -0.078 | <0.001 |  | -0.142 | -0.165, -0.119 | <0.001 |  | -0.043 | -0.064, -0.022 | <0.001 | 43.4% |
| Abstract reasoning | -0.631 | -0.710, -0.552 | <0.001 |  | -0.605 | -0.690, -0.520 | <0.001 |  | 0.025 | -0.027, 0.078 | 0.343 | 4.0% |
| Phonemic verbal fluency | -0.223 | -0.260, -0.186 | <0.001 |  | -0.223 | -0.262, -0.184 | <0.001 |  | 0.0001 | -0.031, 0.031 | 0.994 | 0.04% |
| Semantic verbal fluency | -0.166 | -0.200, -0.133 | <0.001 |  | -0.156 | -0.192, -0.120 | <0.001 |  | 0.010 | -0.018, 0.039 | 0.675 | 6.0% |

^a^ People who retired due to health reasons/ with depression symptoms at retirement, or with only one timepoint measure of cognition before and after retirement, or unemployed/ other before retirement were excluded. Adjusted for retirement age, birth cohort, highest educational qualification, gender, practice effects, spouse employment status, employment grades, still working in the civil service, job demands, job decision latitude, smoking status, alcohol consumption, depressive symptoms, systolic blood pressure, diastolic blood pressure, body mass index, total blood cholesterol, coronary heart disease, stroke, malignant cancers, and diabetes/intermediate hyperglycaemia.

^b^ Calculated as ‘slope after retirement’ minus ‘slope before retirement’.

^c^ Calculated as ‘slope change’ divided by ‘slope before retirement’ and multiplied by 100%.

**Table 3S. Changes of cognition trajectories before and after retirement from sensitivity analysis 2** **(n=3,691) ^a^**

|  | **Before retirement** | | |  | **After retirement** | | |  | **Change ^b^** | | | |
| --- | --- | --- | --- | --- | --- | --- | --- | --- | --- | --- | --- | --- |
|  | **Slope**  **(change per year)** | **95%CI** | ***p*** |  | **Slope**  **(change per year)** | **95%CI** | ***p*** |  | **Slope**  **(change per year)** | **95%CI** | ***p*** | **%**  **change ^c^** |
| Verbal memory | -0.100 | -0.120, -0.081 | <0.001 |  | -0.139 | -0.159, -0.119 | <0.001 |  | -0.038 | -0.058, -0.018 | <0.001 | 38.0% |
| Abstract reasoning | -0.454 | -0.519, -0.389 | <0.001 |  | -0.405 | -0.478 -0.332 | <0.001 |  | 0.048 | -0.014, 0.111 | 0.130 | 10.6% |
| Phonemic verbal fluency | -0.191 | -0.223, -0.160 | <0.001 |  | -0.186 | -0.220, -0.152 | <0.001 |  | 0.005 | -0.025, 0.035 | 0.750 | 2.6% |
| Semantic verbal fluency | -0.155 | -0.183, -0.126 | <0.001 |  | -0.145 | -0.176, -0.114 | <0.001 |  | 0.009 | -0.019, 0.038 | 0.519 | 5.8% |

^a^ Eligible participants but without cognitive function at least once before and once after retirement were included, and missing cognition outcomes were multiply imputed. Adjusted for retirement age, birth cohort, highest educational qualification, gender, practice effects, spouse employment status, employment grades, still working in the civil service, job demands, job decision latitude, smoking status, alcohol consumption, depressive symptoms, systolic blood pressure, diastolic blood pressure, body mass index, total blood cholesterol, coronary heart disease, stroke, malignant cancers, and diabetes/intermediate hyperglycaemia.

^b^ Calculated as ‘slope after retirement’ minus ‘slope before retirement’.

^c^ Calculated as ‘slope change’ divided by ‘slope before retirement’ and multiplied by 100%.

**Table 4S. Changes of cognition trajectories before and after retirement from sensitivity analysis 3 (n=3,433) ^a^**

|  | **Before retirement** | | |  | **After retirement** | | |  | **Change ^b^** | | | |
| --- | --- | --- | --- | --- | --- | --- | --- | --- | --- | --- | --- | --- |
|  | **Slope**  **(change per year)** | **95%CI** | ***p*** |  | **Slope**  **(change per year)** | **95%CI** | ***p*** |  | **Slope**  **(change per year)** | **95%CI** | ***p*** | **%**  **change ^c^** |
| Verbal memory | -0.103 | -0.122, -0.085 | <0.001 |  | -0.142 | -0.162, -0.123 | <0.001 |  | -0.039 | -0.058, -0.021 | <0.001 | 37.9% |
| Abstract reasoning | -0.579 | -0.644, -0.514 | <0.001 |  | -0.547 | -0.616 -0.478 | <0.001 |  | 0.032 | -0.014, 0.079 | 0.176 | 5.5% |
| Phonemic verbal fluency | -0.218 | -0.250, -0.188 | <0.001 |  | -0.216 | -0.249, -0.184 | <0.001 |  | 0.002 | -0.025, 0.029 | 0.860 | 0.9% |
| Semantic verbal fluency | -0.168 | -0.196, -0.139 | <0.001 |  | -0.164 | -0.194, -0.134 | <0.001 |  | 0.004 | -0.022, 0.029 | 0.766 | 2.4% |

^a^ Adjusted for retirement age, birth cohort, highest educational qualification, gender, practice effects, spouse employment status, employment grades, still working in the civil service, job demands, job decision latitude, smoking status, alcohol consumption, depressive symptoms, systolic blood pressure, diastolic blood pressure, body mass index, total blood cholesterol, coronary heart disease, stroke, malignant cancers, diabetes/intermediate hyperglycaemia, and total physical activity.

^b^ Calculated as ‘slope after retirement’ minus ‘slope before retirement’.

^c^ Calculated as ‘slope change’ divided by ‘slope before retirement’ and multiplied by 100%.
